# Unusual one dimensional cascade effect in the thermal and photo-induced switching of azobenzene derivatives on a graphite surface†

**DOI:** 10.1039/d4sc07570f

**Published:** 2025-03-05

**Authors:** Hariom Birla, Showkat H. Mir, Khushboo Yadav, Thomas Halbritter, Alexander Heckel, Jayant K. Singh, Thiruvancheril G. Gopakumar

**Affiliations:** a Department of Chemistry, Indian Institute of Technology Kanpur Kanpur 208016 India gopan@iitk.ac.in +91 5122596830; b Department of Physics, University of Kashmir Srinagar 190006 Jammu and Kashmir India; c Institute for Organic Chemistry and Chemical Biology, Goethe-University Frankfurt Max-von-Laue-Str. 9 60438 Frankfurt Germany; d Department of Chemical Engineering, Indian Institute of Technology Kanpur Kanpur 208016 India; e Center for Nanoscience, Indian Institute of Technology Kanpur Kanpur UP-208016 India

## Abstract

Harnessing cooperative switching opens possibilities for engineering the responses of molecular films to external triggers and provides opportunities to control the directionality of switching/reactions and design novel nanostructures. Here, we demonstrate a one dimensional (1D) cascade effect in the thermal- and photo-induced switching of azobenzene derivatives deposited on a graphite surface. Upon thermal- and photo-induction, molecules switch between their geometric states (*trans* and *cis*) along a selected lattice within the assembly. We explore the switching at the molecular level using atomic force microscopy (AFM) and scanning tunneling microscopy (STM) and reveal that the 1D cascading effect proceeds along the lattice direction where the inter-molecular interaction is the strongest. Theoretical calculations and experiments reveal a cascading effect of up to 350 molecules for photo-induced and 530 molecules for thermal-induced switching along a given lattice.

## Abstract 2

Harnessing cooperative switching opens possibilities for engineering the responses of molecular films to external triggers and provides opportunities to control the directionality of switching/reactions and design novel nanostructures.

## Graphical abstract

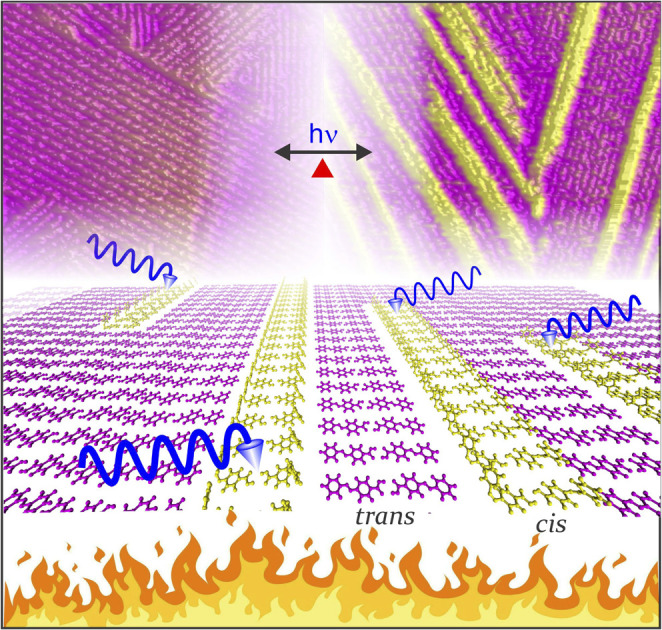

## 1 Introduction

Molecular switches represent a burgeoning field at the intersection of chemistry, materials science, and nanotechnology with promising opportunities in diverse applications such as optical gates for drug delivery,^1–3^ sensors,^4,5^ light responsive materials^6^ and molecular transistors.^4,7,8^ These switches, typically organic molecules, can reversibly change their geometrical states in response to stimuli like light,^9–18^ temperature,^19,20^ electric fields^21–23^ and electrons/holes.^18,24–31^ If these geometric changes can lead to significant changes in the optical/electronic and magnetic properties, then these molecules are considered as molecular switches. Azobenzene (AB) derivatives are among the most important molecules for which *cis*–*trans* switching has been demonstrated on surfaces.^14,17,32^

In supramolecular assembly, it is crucial to control intra- and intermolecular interactions to hinder or enhance the switching efficiency. It has been shown that steric and excitonic coupling can either hinder or enhance switching processes.^33^ Strong surface–molecule interaction may lead to suppression of photoresponse and at the same time an amplification of photo-reaction through cooperativity, where AB molecules support switching in cascades.^34^ Quantitative switching of molecular domains of AB derivatives is driven by intermolecular interactions.^17,35^ Spatial periodicity has been observed in the *trans*–*cis* switching of AB derivatives due to strong molecule–surface interaction and a resultant superlattice.^21^ Due to the difference in the molecule–surface and molecule–molecule interaction, photoswitching of tetra-*tert*-butylazobenzene (TTB-AB) is distinctly different on GaAs and Au(111) surfaces.^36^ On GaAs, a preferential one-dimensional (1D) cascade effect is observed for the switching of TTB-AB.^36^ Cooperative switching occurs when adjacent molecules induce switching in neighboring molecules, leading to synchronized transitions between *trans* and *cis* isomers. This switching effect can amplify the efficiency and response of switching in the solid phase by external stimuli, such as light and/or temperature. Thus, cooperative switching can also give rise to emergent switching behaviors not exhibited by individual molecules in solution. Harnessing cooperative switching opens up possibilities for engineering materials with enhanced responsiveness and improved efficiency and the formation of novel nanostructures.

Cooperativity is a fundamental phenomenon observed in diverse molecular systems, where the interaction between molecules in bulk or two-dimensional assemblies amplifies or diminishes a chemical/physical process. In spin crossover (SCO) systems, if strong cooperativity due to intermolecular interaction exists between neighboring molecules, leading to abrupt spin-state transitions, bistability and hysteresis of spin states are achieved by temperature or pressure.^37,38^ Similarly, hemoglobin exemplifies positive cooperativity in biology, where oxygen binding to one subunit enhances the affinity of other subunits, ensuring efficient oxygen transport.^39–41^ Cooperativity between chromophores can significantly enhance the two-photon absorption cross-section, amplifying nonlinear optical responses for imaging and photodynamic therapy applications.^42–44^ Cooperative switching has also been demonstrated in single molecules^45^ and assembly of molecules.^36^ These examples highlight the universal significance of cooperativity in improving functionality and efficiency across biological, chemical, and material systems. So far, 1D cooperative switching along a given lattice direction in molecular self-assembly is only observed over a few nanometers (nm).^36^

**Fig. 1:**
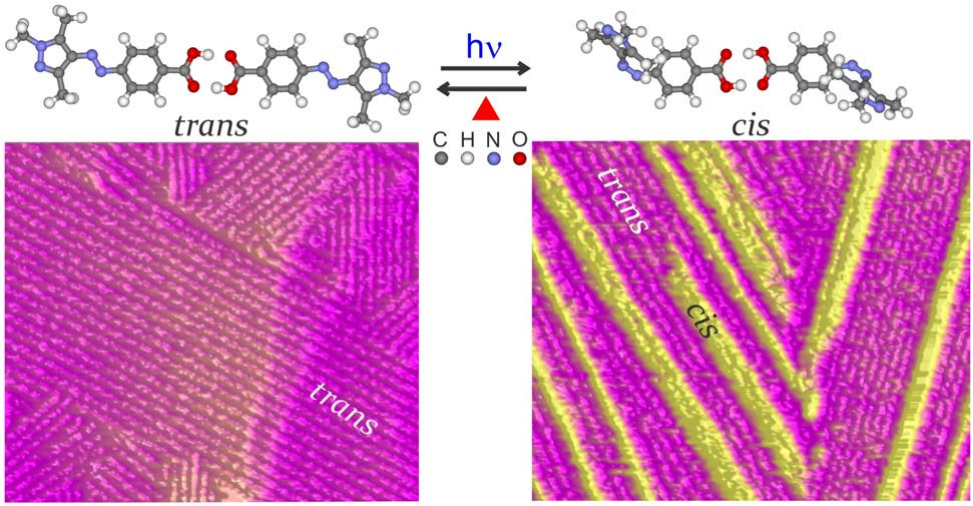
Top panel: optimized geometry of the hydrogen bonded dimer of *trans* and *cis* isomers of PyABA. *Trans* isomer in the PyABA adlayer is switched to the *cis* isomer by photons and are switched back thermally. Lower left panel shows the AFM phase image of the *trans* dominant PyABA adlayer. Lower right panel shows the AFM phase image after illuminating the *trans* dominant PyABA adlayer. Yellow rod-like contrasts correspond to 1D chains of the *cis* isomer within the assembly of *trans* isomers (magenta).

In this article, we show a 1D cascading effect and cooperative *trans*–*cis* and *cis*–*trans* switching over several hundred nm within the adlayers of 4-(2-(2,4-dioxopentan-3-ylidene)hydrazinyl)benzoic acid (PyABA) and 4-(phenylazo)benzoic acid (PABA) molecules at a highly oriented pyrolytic graphite (HOPG)–air interface. The top panel of Fig. 1 shows the schematic of *trans* ↔ *cis* switching of the dimer of PyABA. Due to strong anisotropy in the inter-molecular interaction along different lattices in the adlayer of PyABA and PABA, a strong 1D cascading effect is observed during photo- and thermal-induced switching. The cascading effect extends to several hundred molecules along one of the molecular lattices. The lower panel of Fig. 1 shows the AFM phase images of 1D chains of *cis* isomers within the adlayer of the *trans* isomer of PyABA. The cooperative switching is triggered along the lattice, which has the strongest inter-molecular interaction between the adjacent molecules. The cascading effect is stronger for PyABA than for PABA, which we attribute to the difference in the inter-molecular interactions. It is also observed that the thermal-induced switching has a much stronger 1D cascading effect than photo-induced switching, which is attributed to the involvement of phonon modes.

## 2 Results and discussion

**Fig. 2:**
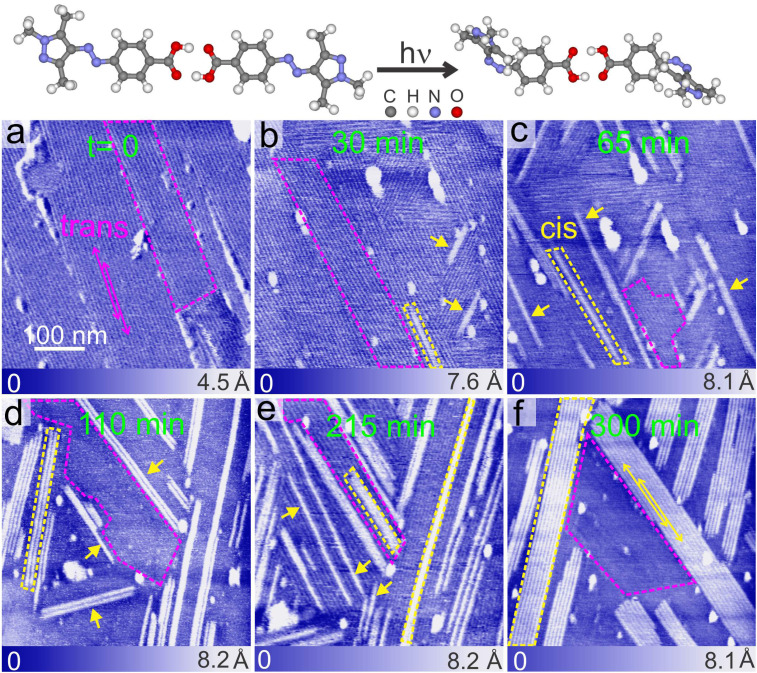
Top panel: optimized geometry of the hydrogen bonded dimer of *trans* and *cis* isomers of PyABA and its photo-induced switching from *trans* to *cis* isomer. (a) Typical AFM topography images of the as-prepared (by dropcasting from methanolic solution) adlayer of *trans*-PyABA on HOPG under ambient conditions. (b–f) Typical AFM topography images of the *trans*-PyABA adlayer after UV illumination (365 nm). The corresponding illumination time is indicated in the image in minutes (min). Magenta and yellow dashed lines indicate the domains corresponding to *trans* and *cis* PyABA, respectively. Yellow arrow heads indicate isolated 1D molecular chains of *cis* isomers. Scale bars of all images are the same (100 nm). Magenta and yellow double headed arrows indicate line-like contrasts corresponding to superlattices in *trans* and *cis* adlayers, respectively.

**Fig. 3:**
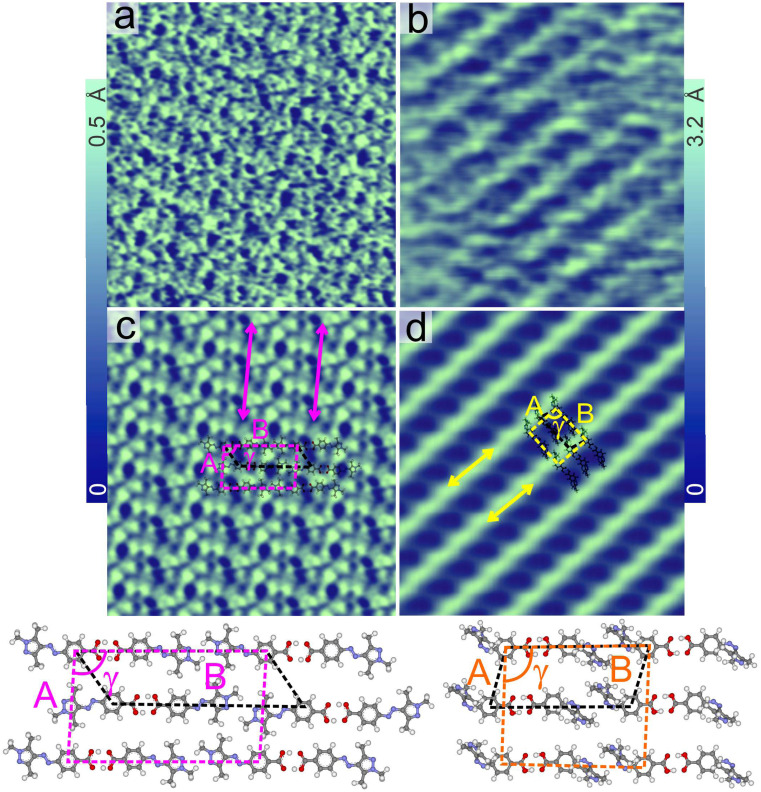
Typical constant current STM image of adlayers (prepared by dropcasting from methanolic solution) of *trans* (a) and *cis* (b) PyABA on HOPG under ambient conditions. The *cis* adlayer is prepared using a solution of PyABA illuminated with UV radiation. Mesh averaged STM images of adlayers of *trans* (c) and *cis* (d) PyABA. Magenta and yellow obliques indicate the unit cells of *trans* and *cis* adlayers, respectively. Double headed arrows indicate the dimer row direction. **A**, **B** and *γ* are the unit lattice parameters. Black dashed obliques represent the smallest cell used for the optimization.

Fig. 2a shows a typical AFM topography image of the adlayer of the *trans* isomer (see in the top panel of Fig. 2) of PyABA with ≈1 monolayer (ML) coverage. The surface is fully covered by mono-crystalline domains (marked with magenta dashed lines) of the *trans* isomer. The domains are observed as long regions, suggesting one dimensional selectivity in the growth. The domains have well defined orientations, typically six (as pairs of three), suggesting the influence of graphite lattice symmetry in the growth of the domains. The line-like features (marked by double headed magenta arrows) are due to a moiré pattern corresponding to the formation of a superlattice.^18,46^ The average distance between the line-like features within the pure adlayer of the *trans* isomer of PyABA is 6.6 ± 0.2 nm. Carboxyl groups are well known for forming robust hydrogen-bonded dimers,^18,46–48^ and we suggest that the building blocks of the adlayer of *trans* PyABA are possibly dimers. We have previously shown that the adlayer of *trans* PyABA is stabilized by dimeric hydrogen-bonding through the carboxyl group.^18,31^Fig. 3a shows the STM topography measured on the adlayer of *trans* PyABA. DFT optimized adlayer of the *trans* isomer is overlaid on the averaged STM topograph in Fig. 3c and a magnified model is shown in the lower panel of Fig. 3. The unit cell of the adlayer of the *trans* isomer is depicted by dashed magenta obliques with **B** (3.0 ± 0.1 nm) and **A** (1.6 ± 0.04 nm) as unit lattice vectors and *γ* as the angle between them. The dimer row is along the **A** lattice direction and is marked by double headed magenta arrows. We also suggest that the *trans* isomer of PyABA lies parallel to the surface plane, and the assembly is stabilized by hydrogen bonded dimer rows. The Moiré pattern is observed generally for the incommensurate lattice and is caused when there is a mismatch between the symmetry of the molecular lattice and surface lattice. The distance of the moiré pattern corresponds to two times the spacing of dimer rows, which suggests that every second adjacent dimer row has a similar adsorption geometry with respect to graphite. To summarize, the line-like features are along one of the lattice directions (**A**) and the hydrogen-bonded dimer rows (marked by the double-headed magenta arrows) of the adlayer.^31^

To understand the switching from the *trans* to the *cis* isomer on the surface (depicted in the top panel of Fig. 2), we illuminated the surface with UV radiation (365 nm) in successive intervals. Fig. 2b–f show typical AFM topographs obtained at different successive illumination times (illumination times are given in the figure). As the time of illumination increases, we observe bright long stripes within the *trans* domains and a few are marked by yellow dashed lines. We refer to these stripes as one dimensional (1D) molecular chains. A few isolated 1D molecular chains are indicated by yellow arrow heads. 1D molecular chains are molecular sized protrusions with an apparent height of ≈5 Å higher than *trans* domains (see the height profile in ESI S1†). This height difference matches approximately the difference in the geometrical height of isomers of PyABA on the surface. This confirms that the long 1D molecular chains are most likely arrays or lattices of *cis* PyABA within *trans* domains. The side view of the density functional theory (DFT) optimized geometry of *cis* and *trans* PyABA layers on bilayer graphite is shown in ESI S2.†

As the time of illumination increases, the following observations are made in the AFM images. The length of the 1D molecular chains increases and reaches a maximum length of ≈1700 nm (300 minutes of illumination). Typically, the 1D molecular chains emerge as individual chains at lower illumination times (*cf.*Fig. 2b–e). The individual 1D chains are indicated by yellow arrow heads. At high illumination (above 150 minutes, *cf.*Fig. 2e and f and ESI S3†), the number of 1D chains rapidly increases and large domains of *cis* PyABA emerge. The statistics of the length of 1D chains and the number of 1D chains as a function of illumination time are provided in Fig. 5a and b, respectively. All observed lengths of 1D chains from multiple areas are included in the statistics. Notably, within just 30 min of illumination, a substantial number of 1D chains are formed with the longest chain length being ≈265 nm (≈350 molecules). This indicates a rapid transition of a substantial number of molecules from the thermodynamically stable *trans* isomer to the *cis* isomer. Interestingly, the 1D chains emerge along the dimer row direction within the adlayer of *trans* PyABA. This implies strong cooperativity in the switching mechanism of molecules along a given lattice, namely along the dimer row (**A** lattice direction). This observation shows that the switching of one molecule induces switching in neighboring molecules along the dimer row. The minimum length of the 1D chains is seemingly comparable for all illumination times and is ≈71 nm. However, the maximum length of the 1D chain systematically increases till 155 min of illumination (see the exponential fit shown using a green line in Fig. 5a). The abundance of isolated 1D chains at all illumination indicates that new 1D chains emerge continuously. The increase in the maximum length of 1D chains suggests that while new 1D chains emerge, the existing 1D chains extend their length further. The value corresponding to short 1D chains must be related to the average “induction length” for a given molecule, which should be related to the cooperativity and strength of intermolecular interaction along the dimer row direction. The “induction length” would be different for different molecules.

To confirm the cooperative switching mechanism along the dimer row, we systematically counted the individual 1D chains as a function of illumination time. A histogram of the number of 1D chains with neighboring 1D chains is shown in Fig. 5b. “One” (black bar) represents the number of 1D chains without neighbors (isolated 1D chain). At low illumination time, the number of 1D chains without neighbors is the maximum. Also, at higher illumination times, there are a substantial number of 1D chains without neighbors. This suggests that the switching of PyABA from its *trans* to *cis* isomer cascades anisotropically along one of the lattices and the switching in one chain is not affected by neighboring 1D chains. Further, the length of the 1D chains shows that the switching is highly cooperative. As the time of illumination increases, the number of 1D chains with neighbors increases (as evident from the high number of 1D chains with two, three, four, or more 1D chains). It is to be noted that above 215 min of illumination, we observe large domains of the *cis* isomer and individual 1D chains are reduced substantially. This shows that the amount of the *cis* isomer on the surface is sufficiently large and rearranges into well defined *cis* domains. It is intuitive that when a high number of adjacent 1D chains of *cis* isomers are available on the surface, they rearrange and form homo-isomer domains for efficient packing. This is also evident in the maximum chain length of 1D chains, which drastically increases after 215 minutes of illumination. After 215 minutes (indicated by red dashed lines), the trend of the maximum chain length does not follow the systematic exponential increase shown by the green line in Fig. 5a. The full scale of data is shown in Fig. ESI S3.† This indicates that the formation of *cis* isomers on the surface follows different kinetics at short and long illumination times. At shorter illumination times, the switching is purely 1D in nature and the 1D chains are formed independently. As the illumination time increases, more and more 1D *cis* chains arise on the surface and they interact with each other and, therefore, form equilibrium domains of *cis* isomers. We also note that illumination times above 300 min up to several hundred min show no measurable change in the size of the domains and the length of the 1D chains in the domains (*cf.* Fig. ESI S4†). However, the possibility of more molecules switching to *cis* for very long illumination times is not ruled out completely.

**Fig. 4:**
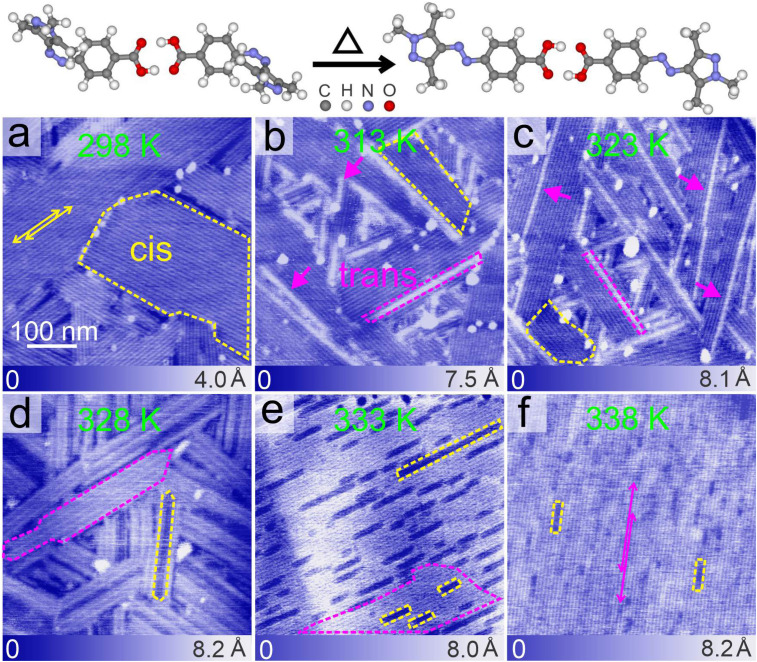
Top panel: optimized geometry of the hydrogen bonded dimer of *cis* and *trans* isomers of PyABA and its thermal-induced switching from the *cis* to the *trans* isomer. (a) Typical AFM topography image of the adlayer of PyABA prepared by dropcasting from a methanolic solution of PyABA on HOPG under ambient conditions. The solution was illuminated with UV radiation before dropcasting. Yellow dashed line indicates a *cis* domain. (b–f) Typical AFM topography images of the *cis*-PyABA adlayer after heating the sample to higher temperatures. Corresponding temperature is indicated in the image. Magenta dashed lines indicate the 1D molecular chain of the *trans* isomer within the adlayer of *cis* PyABA. Magenta arrow heads indicate isolated 1D molecular chains of *trans* isomers. Scale bars of all images are the same (100 nm). Yellow and magenta double headed arrows indicate line-like contrasts corresponding to superlattice in *cis* and *trans* adlayers, respectively.

To understand the microscopic structure of the 1D chains at low illumination time and the domains of the *cis* isomer at high illumination time, we calculated the distance between the adjacent 1D chains and line-like features, respectively. The distance between the 1D chains at short illumination time (≈6.5 nm) is found to be comparable with the distance between the line-like features observed in the *trans* adlayer. Since 1D chains of *cis* isomers emerge within the *trans* adlayer, this is not unexpected. This observation is mainly for those 1D chains with only a few neighboring chains. As mentioned above, at long illumination times, the 1D chains of the *cis* isomers rearrange into pure domains of *cis* isomers. Interestingly, the distance between the line-like features measured in the pure domains of the *cis* isomer is ≈7.1 nm, which is distinctly different from that in the adlayer of *trans* isomers. This clearly indicates that the microscopic structure of the domains of the *cis* isomers is distinctly different from that of the adlayer of the *trans* isomers. To understand this further, we have deposited a *cis* dominant solution of PyABA (by illumination of a methanolic solution of PyABA with a 365 nm LED for about 10 min) on a graphite surface. The adlayer, which is formed by dropcasting the *cis* dominant solution of PyABA, is shown in Fig. 4a. The surface is covered by ≈80% *cis* isomers (see additional images in ESI S5†). The domains of the *cis* isomers are indicated by yellow dashed lines. High resolution images resolve line-like features, originating from the superlattice, within the domains. The distance between the line-like features within the domains of the *cis* isomer is ≈7.1 nm. The correlation between the line-like features in the *cis* domains after on-surface switching (after long illumination of UV) and those in the adlayer prepared after irradiation of the solution suggests that the microscopic structures of both *cis* adlayers are comparable. Thus, we conclude that an equilibrium phase of the *cis* isomer is formed at illumination times longer than 215 min.

The STM topographs recorded for the *cis* adlayer (obtained by switching in solution) reveal the details of the microscopic arrangement of the *cis* isomers within the adlayer and are shown in Fig. 3b. DFT optimized *cis* isomers are overlaid on the averaged STM image (Fig. 3d) and the microscopic arrangement reveals a dimer row based pattern similar to that of the adlayer of the *trans* isomer. The dimers are stabilized by dimeric hydrogen bonding interaction and the dimer rows are indicated by yellow double headed arrows.^18,31^ The unit cell of the adlayer of the *cis* isomer is depicted by dashed yellow obliques with **B** (2.5 ± 0.1 nm) and **A** (1.5 ± 0.04 nm) as unit lattice vectors and *γ* as the angle between them. The dimer row is along the **A** lattice direction. The length of the *trans* and *cis* dimers corresponds to the magnitude of the **B** vector and is 3.0 ± 0.1 nm and 2.5 ± 0.1 nm, respectively. Thus, the superlattice distance of the *trans* and *cis* adlayers is also expected to be different, as observed in the AFM images.

After establishing the photo-induced switching of PyABA molecules from the *trans* to the *cis* isomer that proceeds in a one-dimensional manner along a selected lattice, we investigated the on-surface *cis* to *trans* switching. As established in solution, *cis* to *trans* switching can be induced by temperature.^20,49^ We adapted temperature-induced switching for the on-surface *cis* to *trans* switching. The prepared adlayer shown in Fig. 4a is further annealed to higher temperatures in an interval of ≈5 K. Fig. 4d–f show the AFM topography images of the successively heated adlayer. As shown above, the surface is populated with ≈80% *cis* isomer (*cf.*Fig. 4a and ESI S5†). The population of *cis* isomers on the surface matches well with the population of *cis* isomers in UV irradiated solution (see the Materials and methods). The *cis* domains are marked with yellow dashed lines. Since the lifetime of the *cis* isomer of PyABA is several thousand days,^50,51^ spontaneous conversion from *cis* to *trans* on the surface at room temperature is not expected in the framework of our experimental time.

**Fig. 5:**
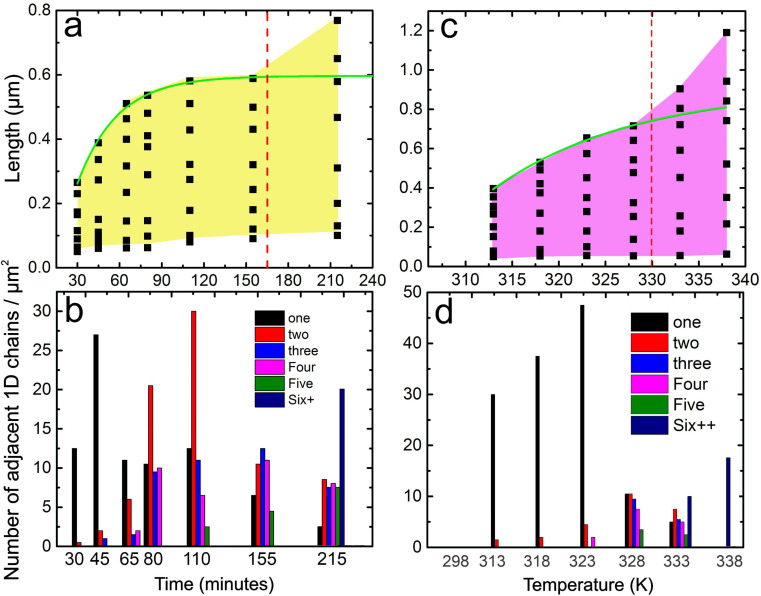
The statistics of the length of 1D *cis* chains (a) and 1D *trans* chains (c) for photo- and thermal-induced switching in PyABA adlayers, respectively. The statistics of the number of 1D *cis* chains (b) and 1D *trans* chains (d) for the photo- and thermal-induced switching in PABA adlayers, respectively. Black bar (“one”) depicts the number of 1D chains with no neighbors. Green solid lines depict exponential fits to the maximum length of 1D chains. Red dashed line depicts the illumination time/temperature from which the exponential fit deviates.

As the temperature increases (*cf.*Fig. 4b and c), long 1D stripes start appearing within the *cis* domains, which are marked by magenta dashed lines. We attribute these 1D stripes to the switching of *trans* isomers within the *cis* domains and are termed 1D *trans* chains. A schematic of the *cis* to *trans* switching is shown in the top panel of Fig. 4. The corresponding statistical analysis of the length of 1D *trans* chains and the number of neighboring chains is shown in Fig. 5c and d, respectively. The 1D *trans* chains are as long as 395 nm at lower annealing temperature (313 K) and increase systematically to 715 nm at 328 K. Similar to the photo-induced *trans* to *cis* switching, the *cis* to *trans* switching shows an exponential increase (indicated using the green curve in Fig. 5c) in the length of the 1D *trans* chains. Above 328 K, the chain length increases rapidly and reaches up to 1200 nm (338 K). Within the range of 313 to 328 K, the chains notably emerge as individual chains. A few individual chains are also indicated by magenta arrow heads. This observation is striking and is similar to that observed for the on-surface *trans* to *cis* switching. We also note that the 1D chains emerge along the dimer row of the assembly, which also corresponds to one of the lattices (**A**, *cf.*Fig. 3b). The 1D anisotropy in the switching suggests strong cooperativity between the molecules along the **A** lattice. This shows that the switching of a dimer to the *trans* isomer induces the neighboring dimers along the **A** lattice to also switch to the *trans* isomer. No switching is induced along the **B** direction, as evident from the individual 1D chains. Till 328 K (Fig. 4c and d), 1D *trans* chains emerge individually and the length of the chains increases systematically. Above 328 K (Fig. 4e and f), the surface is significantly covered by the *trans* isomers and the remaining *cis* isomers are observed as distinct 1D chains (marked by yellow dashed lines). This further re-establishes that at each stage of annealing, the switching of *cis* to *trans* is 1D in nature and is strictly along the **A** direction. We presume that above 328 K, there are a sufficient number of *trans* isomers on the surface, which form their equilibrium domains. This is also evident in the drastic increase of the maximum chain length of 1D *trans* chains above 328 K (marked by the red dashed line). Above 328 K, the chain length does not follow the systematic increase marked by the green dashed line in Fig. 5b. The minimum length of 1D chains is comparable at all annealing temperatures and is ≈54 nm, which is slightly lower compared to that of the minimum chain length observed in the photo-induced switching. Thus, we suggest the “induction length” to be characteristic of the isomers of the molecule.

The 1D nature of switching is further supported by the number of 1D *trans* chains as a function of temperature (*cf.*Fig. 3d). The black bar (“one”) depicts the number of 1D chains with no neighbors. Till 323 K, the majority of the 1D chains have no neighbors and support the strong anisotropy in the *cis* to *trans* switching. Beyond 323 K, the count of 1D chains with no neighbors decreases drastically. Due to the emergence of more and more chains, the *trans* isomers form pure *trans* domains at higher annealing temperatures. As evident from the images, large uniform *trans* domains are observed (*cf.*Fig. 4e and f). The distance between the line-like features in the *trans* domains (≈6.5 nm) that emerged after annealing to higher temperatures corresponds to that observed in Fig. 2a. This suggests that after annealing to 338 K, the *cis* domains are fully converted to the equilibrium microscopic structure of the *trans* adlayer. Heating beyond 338 K leads to desorption of PyABA molecules from the surface.

**Fig. 6:**
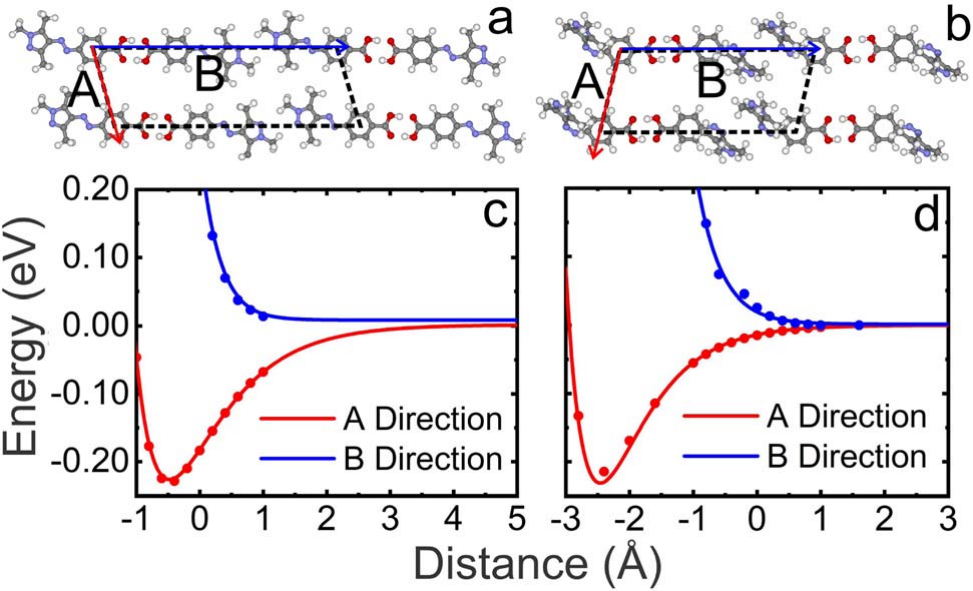
DFT optimized geometry of the smallest unit cell for *trans* (a) and *cis* (b) adlayers of PyABA on bilayer graphite. **A** and **B** are the unit lattice parameters. The interaction energy profile (PES) of adjacent dimers along the **A** (red dots) and **B** (blue dots) directions in *trans* (c) and *cis* (d) adlayers of PyABA. The interaction energy is calculated without bilayer graphite. The full line curve depicts the corresponding Morse potential. Zero distance marks the optimized distances of **A** and **B** on bilayer graphite.

The prima facie argument for the 1D cascading effect in the photo-induced *trans* to *cis* and the thermal-induced *cis* to *trans* switching of PyABA molecules on the surface is a strong cooperativity between them. The cooperativity must be directly linked to the intermolecular interactions within the *trans* and *cis* adlayers. The dots in Fig. 6c and d show the interaction energy profile (potential energy scan, PES) between adjacent dimers along two compact lattice directions (**A** and **B**) within the *trans* and *cis* adlayers, respectively. Further, the scan is fitted with a Morse potential (red and blue lines) as expected for a typical PES. The PES calculations are performed using the optimized unit cell shown in Fig. 6c and d, respectively. The unit cell is optimized on bilayer graphite for a commensurate lattice; however, the scan is performed without graphite. Zero distance in the scan depicts the optimized value of **A** and **B** on graphite. Remarkably, the calculation reveals strong intermolecular coupling along the **A** direction for both *trans* and *cis* adlayers. The interaction energy is 226 meV and 231 meV along the **A** direction for *trans* and *cis* adlayers, respectively. Conversely, along the **B** direction, the energy steadily escalates as dimers approach and appears as an unbound state. That is, the interaction energy of dimers along the **B** direction is nearly zero. The strong anisotropy in the intermolecular interaction along **A** and **B** directions directly correlated with the direction of 1D switching. Because the intermolecular interactions are stronger along **A**, any change in the state of one molecule within the adlayer induces the neighboring molecule to change its states. Neighbouring molecules in a similar state are energetically more favorable than those different states. Since there is strong anisotropy in the intermolecular interactions along different lattice directions, it is summarized that the anisotropy will influence the induction of the states of neighboring molecules within the assembly as observed in 1D switching.

It should be mentioned that in condensed phases of molecular crystals, phase transitions may be facilitated by low energy collective vibrations (phonon modes) of molecular lattices.^52,53^ The strong intermolecular interaction along the **A** direction in the PABA film may pertain to phonon modes exclusively along this lattice and can facilitate the 1D cascading effect in the switching. It is noted that the maximum length of 1D chains and the number of 1D chains per unit area for thermal-induced *cis* to *trans* switching are higher than those of the photo-induced *trans* to *cis* switching. As temperature could activate phonon modes, a stronger 1D cascading effect is apparent in the thermal-induced *cis* to *trans* switching. While the intermolecular interaction strength is comparable for both *trans* and *cis* adlayers, the increased 1D chain length in the thermal-induced *cis* to *trans* switching supports the influence of phonon modes. Phonon mode along a given crystallographic stacking directly facilitates the phase transition.^53^ We also note that the electronic decoupling of molecules from the graphite surface (unlike on metal surfaces) may also add to the efficiency in the 1D cooperative switching. The above elucidation serves as a pivotal insight into the relationship between molecular interactions within the lattice and the cooperative 1D switching behavior in the photo-induced *trans* to *cis* and the thermal-induced *cis* to *trans* switching in the PyABA adlayer. As per our previous study, using tunneling spectroscopy,^31^ the efficiency of electron/hole induced molecular switching in the *cis* adlayer of PABA is higher by several folds compared to that in the *trans* adlayer. This has been attributed to the difference in the electronic structure of *cis* and *trans* isomers on the surface and the difference in the energy barriers for *cis* to *trans* and *trans* to *cis* switching.^31,54^ Thus, it is concluded that the involvement of phonon modes and the higher efficiency of *cis* to *trans* switching collectively contribute to the formation of longer 1D chains in thermal-induced switching compared to photo-induced *trans* to *cis* switching.

To manifest the above observation of 1D cooperative switching, we have studied the switching in the adlayer of another AB derivative (4-(phenylazo) benzoic acid, PABA). The AFM images of the photo-induced *trans* to *cis* and the thermal-induced *cis* to *trans* switching are provided in ESI S6 and S7,† respectively. The microscopic structure of equilibrium domains of *trans* and *cis* adlayers of PABA is provided in ESI S8.† The building blocks of the adlayer of both *trans* and *cis* isomers are hydrogen bonded dimers similar to that in PyABA. The AFM images reveal 1D cooperative photo- and thermal-induced switching in the *trans* and *cis* adlayer of PABA on graphite. This is revealed through the observation of 1D *cis* and *trans* molecular chains in *trans* and *cis* adlayers, respectively. To further account for the 1D nature of the switching, we have analyzed the statistics of the length of 1D chains and the number of 1D chains with neighbors for the PABA adlayer. The length of all switched 1D *trans* chains is plotted as a function of UV irradiation time and the annealing temperature is provided in ESI S9.† The length of the longest chain (300 nm at 45 minutes) in photo-induced switching increases exponentially (see the green line corresponding to the exponential fit) and reaches up to 1000 nm. The minimum length of the chains (induction length) in the photo-induced switching is ≈80 nm. To further investigate the 1D selectivity, we have analyzed the number of neighboring chains as a function of the illumination time. It is noted that the number of neighboring chains also increases rapidly as the illumination increases, except for very short illumination time. This suggests that the switching progress along both **B** and **A** lattice directions simultaneously. This is further confirmed by the interaction energy profile (PES) of the dimers along the **B** and **A** lattice directions within the *trans* adlayer and is shown in ESI S10.† Compared to PyABA, the interaction energy along **A** is only 17 meV and along **B** is nearly zero. That is, the strength of cooperativity along **A** for PABA is much lower compared to that in PyABA. This is observed as the weak 1D selectivity in the photo-induced switching. In contrast, we observe a strong 1D cascading effect for the thermal-induced *cis* to *trans* switching in the *cis* adlayer of PABA. This is correlated with the strong intermolecular interaction along the **A** lattice direction (162 meV) compared to that along the **B** lattice (nearly zero). Thus, we establish that strong anisotropy in the intermolecular interaction strength along different lattice directions leads to high cooperativity and the 1D cascade effect in the switching appears along the given lattice directions.

## 3 Conclusion

In conclusion, we have studied the thermal- and photo-induced switching of azobenzene derivatives (PyABA and PABA) deposited on graphite surfaces at the molecular level using AFM, STM and DFT calculations. The photo-induced *trans* to *cis* and the thermal-induced *cis* to *trans* switching of these molecules exhibit a strong 1D cascading effect while switching. Several hundred molecules are switched through strong cooperative effects along a given lattice of the assembly. It is revealed that the strong intermolecular interaction along one of the lattices compared to the other is the origin of the 1D cascading effect. We also notice the same anisotropy while the molecular assembly is formed, which is also attributed to strong anisotropy in the intermolecular interactions along different lattice directions. The ability of the molecules to switch in a cooperative manner along a single lattice direction highlights the potential for precise control of chemical reactions or communications in molecular-scale devices. The insight into the 1D cooperative switching processes opens avenues for designing materials and systems with tailored switching properties, essential for the development of advanced nanotechnology and molecular electronics.

## 4 Materials and methods

### 4.1 Experimental details

4-(2-(2,4-Dioxopentan-3-ylidene)hydrazinyl)benzoic acid (PyABA) was synthesized^55^ and, after purification, directly used for the experiments. PABA is purchased from Aldrich with purity above 98%. Methanolic solutions of PyABA and PABA with a concentration of 10^−5^ M were used for the preparation of the adlayer of the *trans* isomer. At thermal equilibrium, the solution of PyABA and PABA contains predominantly the energetically most favorable *trans* isomer under ambient conditions.^18,31^ Methanol of analytical grade (Fisher Scientific) with purity > 99.9% was used. For the preparation of the adlayer of the *cis* isomer, the solutions of PyABA and PABA were illuminated with UV radiation (365 nm LED and an output power of ≈190 mW) for 10 minutes. The population of *cis* isomers of PyABA and PABA in the UV-irradiated solution is obtained from time dependent photo-induced solution state dynamics and is provided in ESI S11.† After illumination with UV, ≈77% and ≈44% of PyABA and PABA, respectively, are converted to their *cis* isomer. After reaching the photo-stationary state (≈10 min), the solutions of PyABA and PABA are immediately drop-cast on a highly oriented pyrolytic graphite (HOPG) 0001 surface and subsequently the surface was dried in a vacuum. The substrate was tilted at ≈30° while drop-casting to ensure a smooth flow of the solution. Relative humidity and temperature were maintained at ≈50% and 22–25 °C, respectively, during the sample preparation and imaging. All microscopic structural analysis on the surface was carried out using an Agilent 5500 atomic force microscope (AFM) and an RHK ATM300 scanning tunneling microscope (STM). AFM/STM imaging was performed around 22–25 °C under ambient conditions. The on-surface switching (*trans* to *cis*) of molecules is performed by illuminating the adlayer of the *trans* isomer with a 365 nm LED and the amount of light on the surface is ≈38 lux. The post-processing of images (AFM) was performed using WSxM from Nanotec.^56^ All experiments are performed on nearly 1 ML coverage. The coverage is confirmed by AFM topography corresponding to the sub-monolayer film (ESI S12†). Using the pristine graphite surface visible between the molecular domains, the coverage is determined. The nature and the microscopic details of molecular islands are comparable for both 1 ML and sub-ML coverage. Note that STM imaging is performed only on the initial *trans*/*cis* adlayer and the final *cis*/*trans* dominant adlayer to understand their microscopic structure.

### 4.2 Computational details

We employed Density Functional Theory (DFT)^57,58^ to investigate the adsorption geometry and the interaction energy between adjacent molecules in adlayers. The exchange–correlation between electrons was treated by generalized gradient approximation (GGA)^59^ using Perdew–Burke–Ernzerhof (PBE)^60^ parametrization and rrkjus^61^ pseudo-potentials. Ionic positions were relaxed using the Broyden–Fletcher–Goldfarb–Shanno (BFGS)^62^ algorithm, with a pressure convergence threshold of 0.5 kbar. For electron relaxation, we set a convergence criterion of 1 × 10^−6^. The Kohn–Sham^63^ states were expanded in a plane wave basis set with a cutoff energy of 50 Ry for the wave function and 400 Ry for charge density. We have also included van der Waals interactions using Grimme's dispersion-correction scheme (D3).^64^ Geometry optimization and self-consistent field (SCF) calculations were performed on a *k*-mesh of 6 × 8 × 1, whereas NSCF calculations were carried out with a Monkhorst–Pack 8 × 10 × 1 *k*-point grid.

To generate the potential energy scan/surface (PES), we focused on a unit cell containing four AB molecules, while excluding the influence of HOPG. We systematically examined the interactions between the AB molecules along both **A** and **B** by incrementally adjusting the inter-molecular distances in finite steps, analyzing both *trans* and *cis* conformations. Initially, we fixed the distance between molecules along the **B** direction at an optimized value while varying the separation along the **A** direction. We computed the self-consistent field (SCF) energy at various distances from this optimized value and plotted the energy as a function of molecular separation. This process was then mirrored for the **B** direction, with the distance along the **A** lattice held constant. An important question arises regarding our choice to omit the graphite effect in deriving the PESs. This decision was made to reduce computational costs and the difficulty in implementing the varying dimension of the unit cell (**A** and **B** are varying in the PES). In addition, we assert that this approach does not compromise the overall conclusions because of weak interaction energy between molecules and graphite.

## Data availability

Part of the data supporting this article have been included in the ESI.† In addition, any data related to the manuscript will be made available upon request.

## Author contributions

HB: photo- and thermal-induced experiments and theoretical and experimental data analysis. SHM: theoretical calculations. KY: STM experiments. TH: synthesis of molecules. AH: supervision of the synthesis of molecules. JKS: supervision of theoretical calculations. TGG: planning and coordinating experiments and theory, overall supervision and compilation of the paper.

## Conflicts of interest

There are no conflicts to declare.

## Supplementary Material

SC-OLF-D4SC07570F-s001
