# Supplementary material for: Unusual one dimensional cascade effect in the thermal and photo-induced switching of azobenzene derivatives on a graphite surface
Source: Chem Sci. 2025 Mar 5;16(15):6325–35. doi: 10.1039/d4sc07570f (PMC11898271; doi:10.1039/d4sc07570f)
Supplement: SC-016-D4SC07570F-s001 [file SC-016-D4SC07570F-s001.pdf]

Supplementary Information (SI) for Chemical Science.
This journal is © The Royal Society of Chemistry 2025

Electronic Supplementary Information (ESI†) Unusual One Dimensional Cascade Effect in the Thermal and Photo-Induced Switching of Azobenzene Derivatives on Graphite Surface

Hariom Birla,† Showkat H. Mir,‡ Khushboo Yadav,† Thomas Halbritter,¶,‖ Alexander Heckel,¶ Jayant K. Singh,§ and Thiruvancheril G. Gopakumar*,†

†*Department of Chemistry, Indian Institute of Technology Kanpur, Kanpur 208016, India*

‡*Department of Physics, University ofKashmir, Srinagar 190006, Jammu and Kashmir*

¶*Institute for Organic Chemistry and Chemical Biology, Goethe-University Frankfurt, Max-von-Laue-Str. 9, 60438 Frankfurt, Germany*

§*Department of Chemical Engineering, Indian Institute of Technology Kanpur, Kanpur 208016, India*

‖*Current Address: WuXi AppTec GmbH, Am Haag 16, Gräfelfing, 82166, Bavaria, Germany*

E-mail: gopan@iitk.ac.in

Phone: +91 5122596830. Fax: +91 5122596806

S1: AFM topographs of UV illuminated adlayer of *trans* PyABA

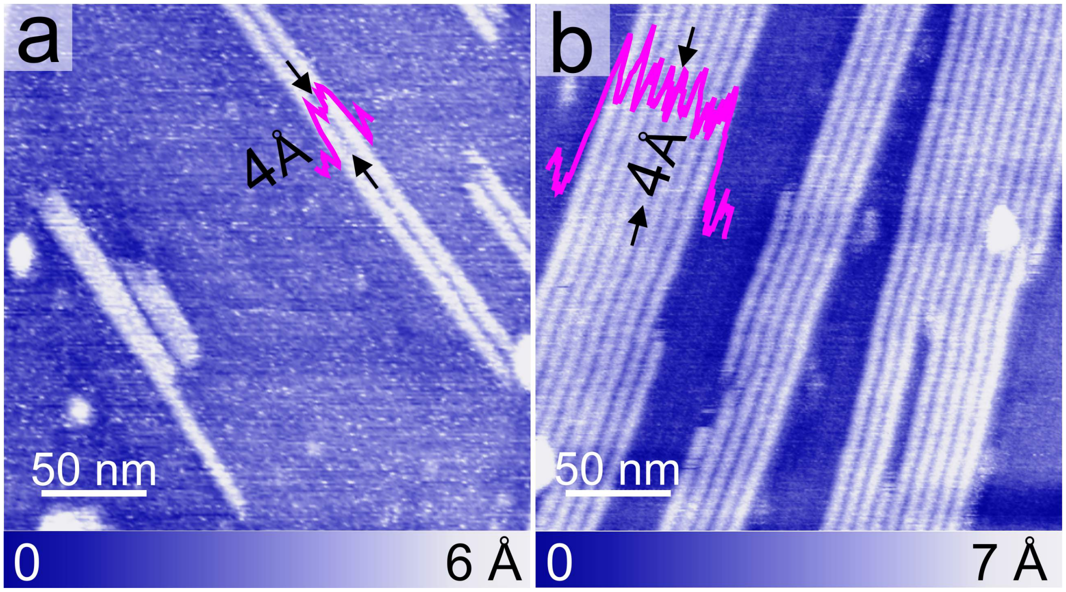

Figure 1: AFM topographs of adlayer of *trans* PyABA on HOPG after illuminated with UV radiation for 110 (a) and 300 (b) minutes. The bright long stripes are 1D *cis* chains formed after UV illumination. The corresponding height profiles of the 1D chains are overlaid. The height of the 1D *cis* chains is ≈ 4 Å with respect to the *trans* adlayer. The height is in accordance to the typical height difference of optimized geometry of adlayer of *cis* with respect to *trans* on bilayer graphite.

S2: Optimized geometry of *trans* and *cis* dimer of PyABA on bilayer graphite.

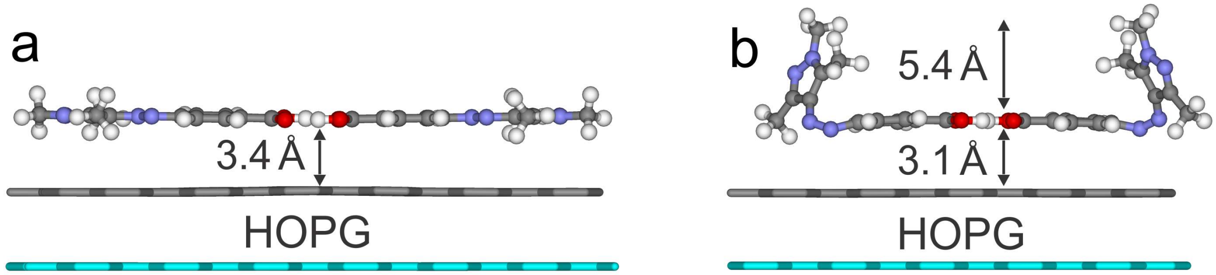

Figure 2: Side view of the optimized geometry of *trans* (a) and *cis* (b) dimer of PyABA on bilayer graphite. The dimers are taken from the fully optimized adlayer of *trans* and *cis* PyABA on bilayer graphite. The average height of the *cis* dimer is 5.1Å higher than the *trans* dimer.

S3: AFM topographs of the adlayer of *trans* PyABA molecule before and after UV irradiation.

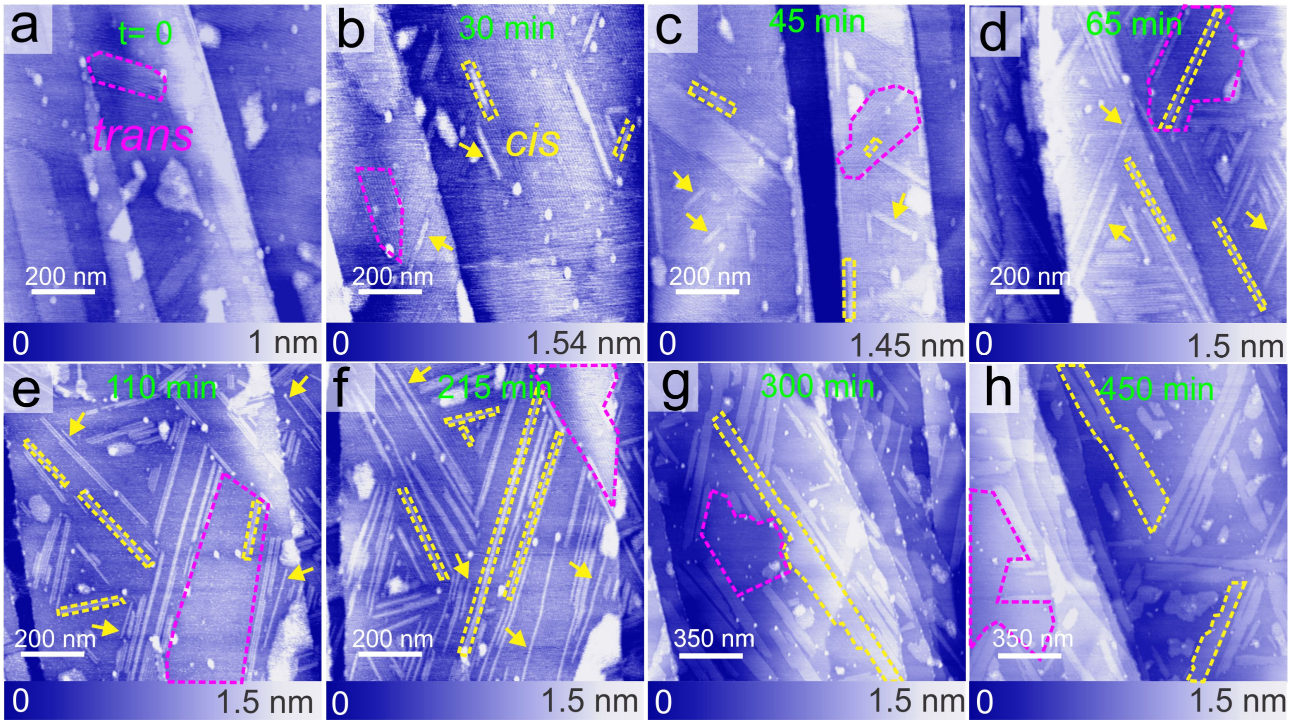

Figure 3: Sequence of AFM topographs of the adlayer of *trans* PyABA on HOPG before (a) and after UV irradiation (b – h) with 365 nm wavelength. The corresponding time of illumination is included in the images. Magenta and yellow dashed lines mark the chains/domains of *trans* and *cis* isomers of PyABA, respectively. Before UV irradiation, the surface is fully covered by *trans* isomers. 1D chains and domains of *cis* isomers are formed by the photo-induced switching of *trans* to *cis*. Till 215 minutes of illumination of UV, the abundance of isolated 1D *cis* chains are high. A few isolated 1D *cis* chains are marked by yellow arrow heads. Further exposure leads to the formation of large *cis* domains of PyABA as the abundance of *cis* isomer increases on the surface.

S4: The length of 1D *cis* chains as a function of UV illumination time.

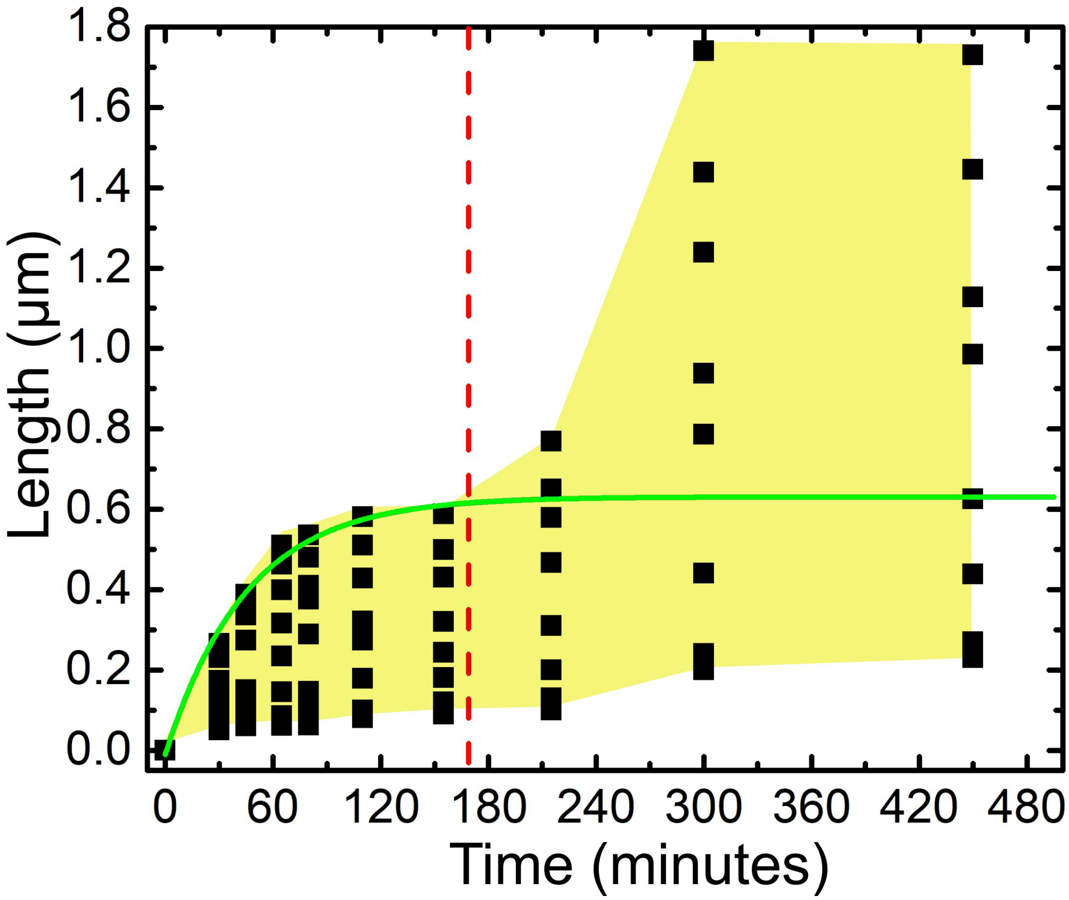

Figure 4: The length of 1D *cis* chains as a function of time of UV illumination on the adlayer of *trans* PyABA. Till 155 min of UV illumination, the maximum chain length increases systematically. Green line depicts an exponential fit of the maximum chain length of 1D *cis* chains. Till 155 min, the exponential fit is correlating with the 1D chain length. After 155 min of illumination, the maximum chain length increases drastically and saturates by 300 min of illumination. This drastic increase is attributed to the formation of large domains of *cis* isomers on the surface.

S5: AFM topographs of the adlayer of *cis* PyABA molecule before and after annealing.

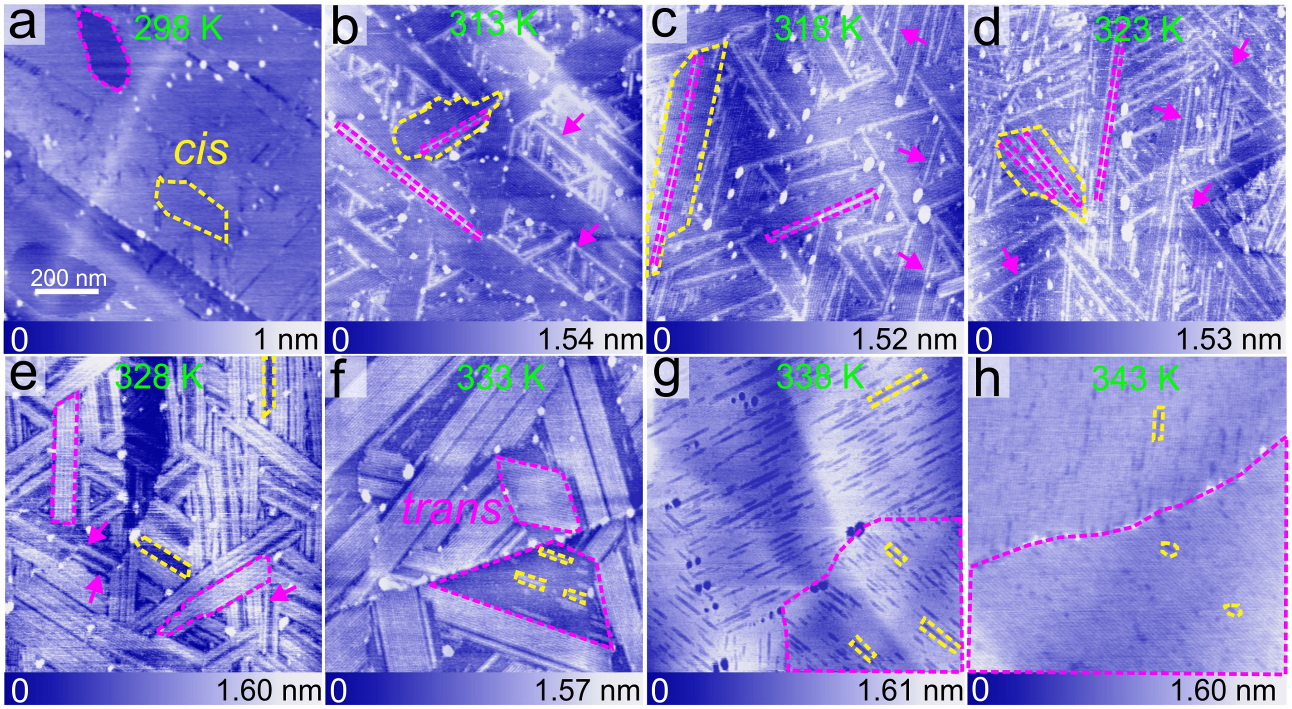

Figure 5: Sequence of AFM topographs of the adlayer of *cis* dominant PyABA on HOPG before (a) and after annealing (b – h). The corresponding annealing temperature is included in the images. Magenta and yellow dashed lines mark the chains/domains of *trans* and *cis* isomers of PyABA, respectively. Before annealing (a), the surface is covered by ≈ 80% *cis* isomers. 1D chains and domains of *trans* isomers are formed by the thermal–induced *cis* to *trans* switching. Till 328 K, isolated 1D *trans* chains are highly abundant. A few isolated 1D *trans* chains are marked by magenta arrow heads. Further annealing above 328 K, leads to the formation of large *trans* domains of PyABA as the abundance of *trans* isomer increases on the surface. It is to be noted that the remaining *cis* isomers within the switched *trans* domains exist as long stripes, indicating the 1D nature of switching even at high annealing temperature. Above 343 K, the *trans* adlayer of PyABA desorbs.

S6: Photo-induced *trans* to *cis* switching of PABA in the adlayer of *trans* isomers.

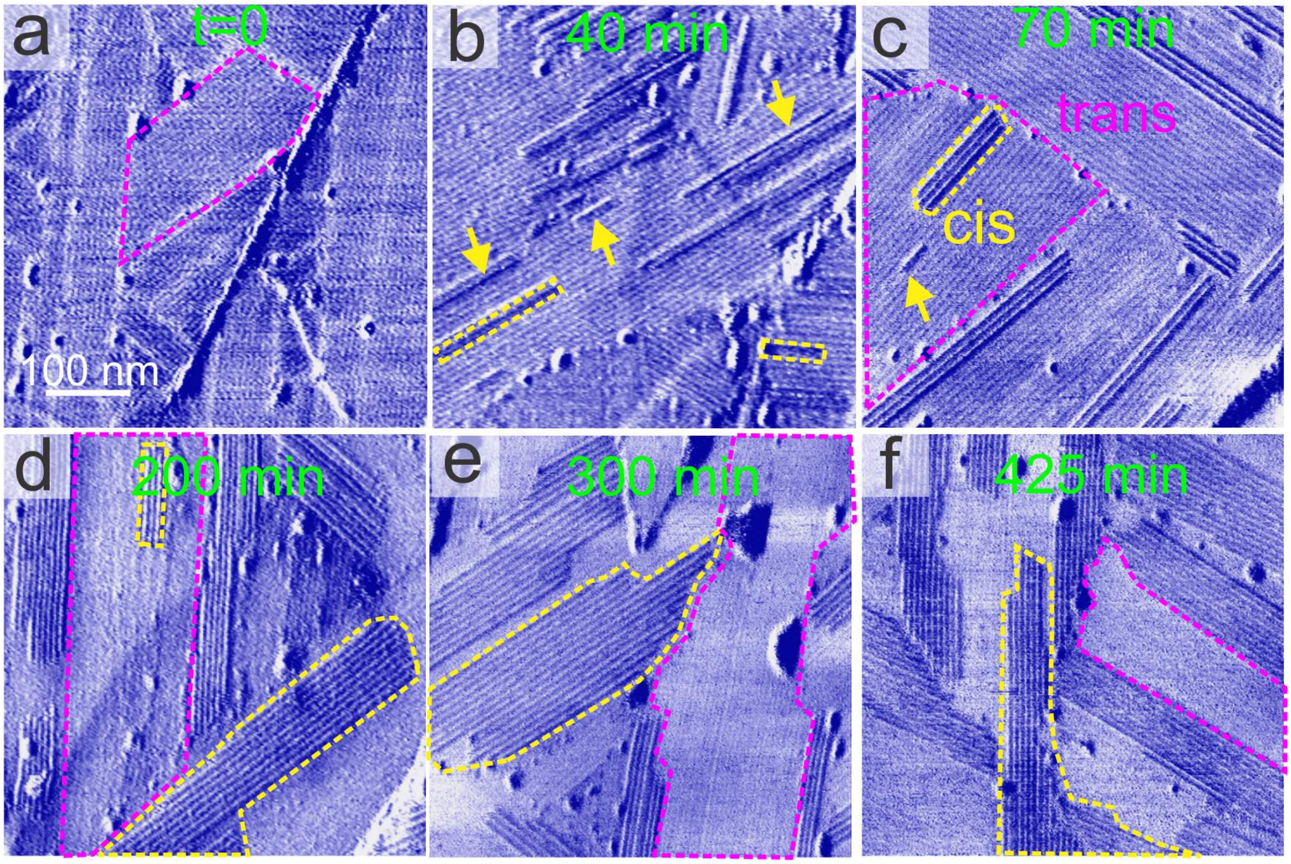

Figure 6: Sequence of AFM phase images of the adlayer of PABA on HOPG before (a) and after UV irradiation (b – f) with 365 nm wavelength. The corresponding time of illumination is included in the images. Magenta and yellow dashed lines mark the chains/domains of *trans* and *cis* isomers of PABA, respectively. Before UV irradiation, the surface is fully covered by *trans* isomers. 1D chains and domains of *cis* isomers are formed by the photo-induced switching of *trans* to *cis*. Unlike PyABA, the 1D selectivity is only visible at low illumination time. A few isolated 1D *cis* chains are marked by yellow arrow heads. Above 70 min of exposure of UV radiation, large *cis* domains of PABA is observed.

S7: Thermal-induced *cis* to *trans* switching in *cis* dominant adlayer of PABA.

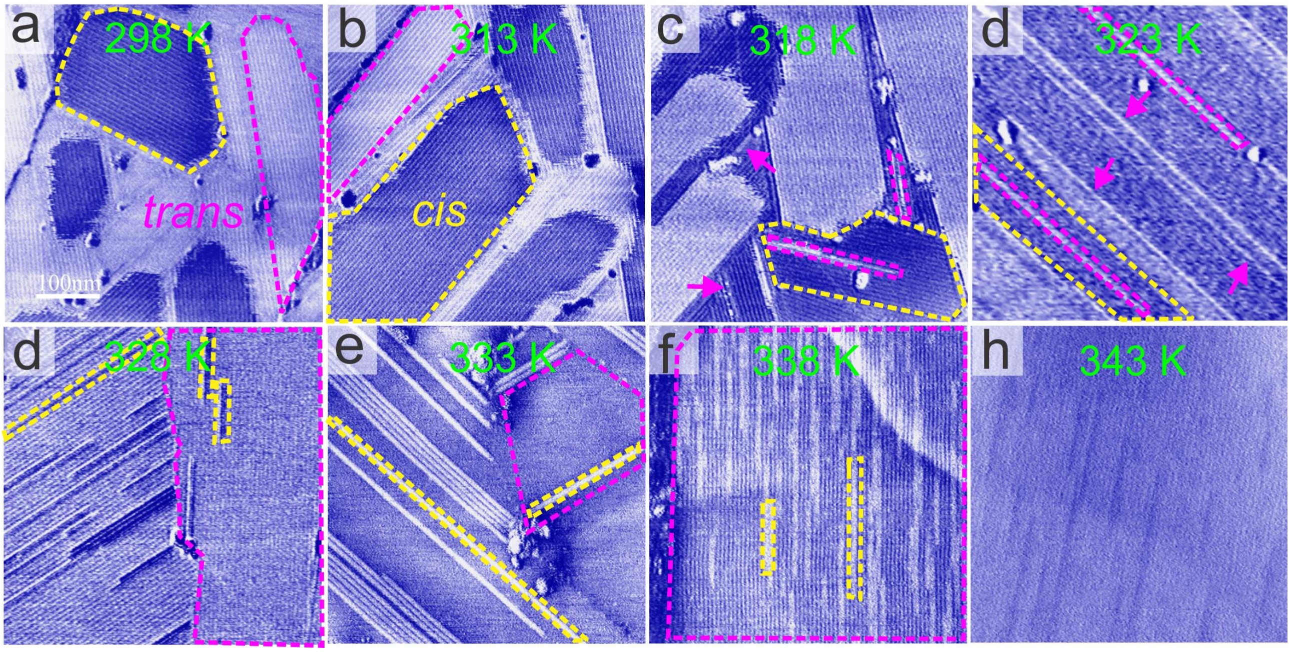

Figure 7: Sequence of AFM phase images of the adlayer of mixture of *cis* and *trans* PABA on HOPG before (a) and after annealing (b – h). The corresponding annealing temperature is included in the images. Magenta and yellow dashed lines mark the chains/domains of *trans* and *cis* isomers of PABA, respectively. Before annealing (a), the surface is covered by $\approx 50\%$ *cis* isomers. 1D chains and domains of *trans* isomers are formed by the thermal–induced *cis* to *trans* switching. Till 328 K, isolated 1D *trans* chains are abundant. A few isolated 1D *trans* chains are marked by magenta arrow heads. Further annealing above 328 K, leads to the formation of large *trans* domains of PABA as the abundance of *trans* isomer increases on the surface. It is to be noted that the remaining *cis* isomers within the switched *trans* domains exist as long stripes indicating the 1D nature of switching even at high annealing temperature. Above 343 K, the *trans* adlayer of PABA desorbs.

S8: STM topographs of adlayer of *trans* and *cis* PABA on HOPG

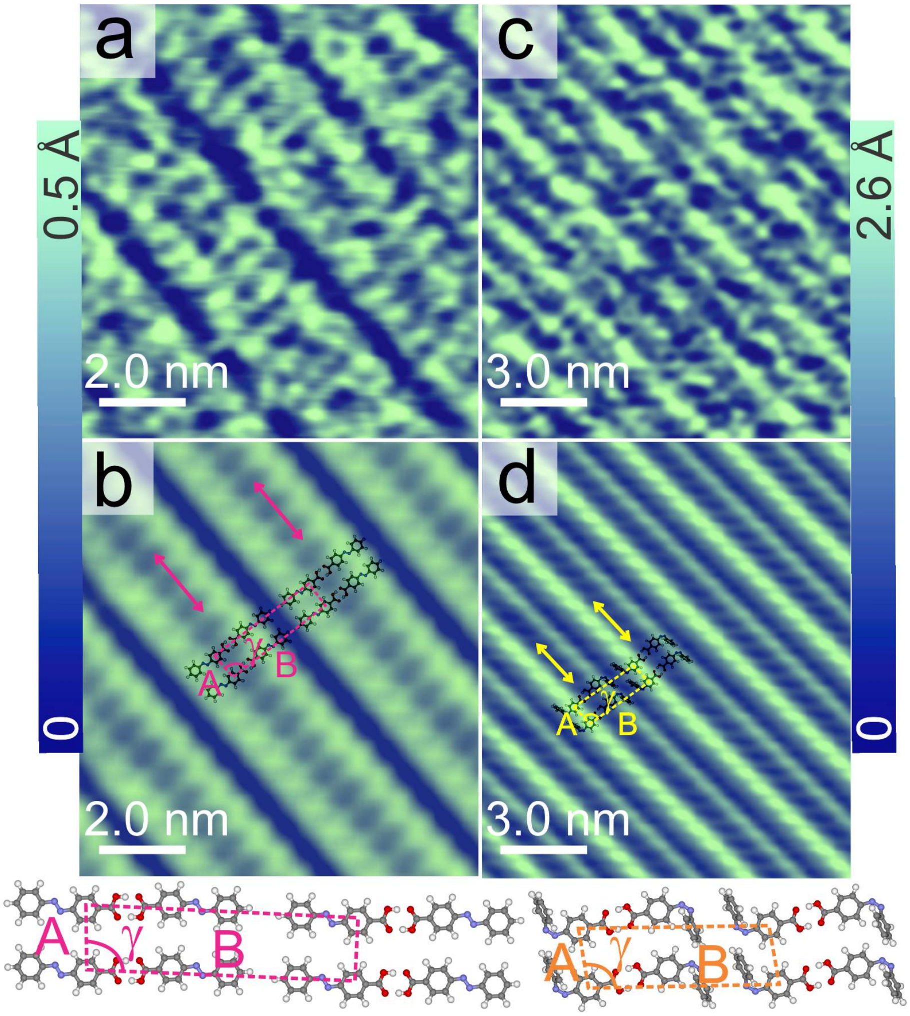

Figure 8: Typical constant current STM image of the adlayer of *trans* (a) and *cis* (c) adlayer of PABA dropcasted from methanol on HOPG under ambient condition. The *cis* adlayer is prepared using a solution of PABA illuminated with UV radiation (365 nm). Mesh averaged images of adlayers of *trans* (b) and *cis* (d) PABA is also shown. Magenta and yellow obliques indicate the unit cells of *trans* and *cis* adlayers, respectively. Double headed arrows indicate the dimer row direction. **A**, **B** and γ are the unit lattice parameters.

S9: The statistics of the length of 1D *trans* chains and the number of neighboring chains for the photo– and thermal–induced switching in PABA adlayers.

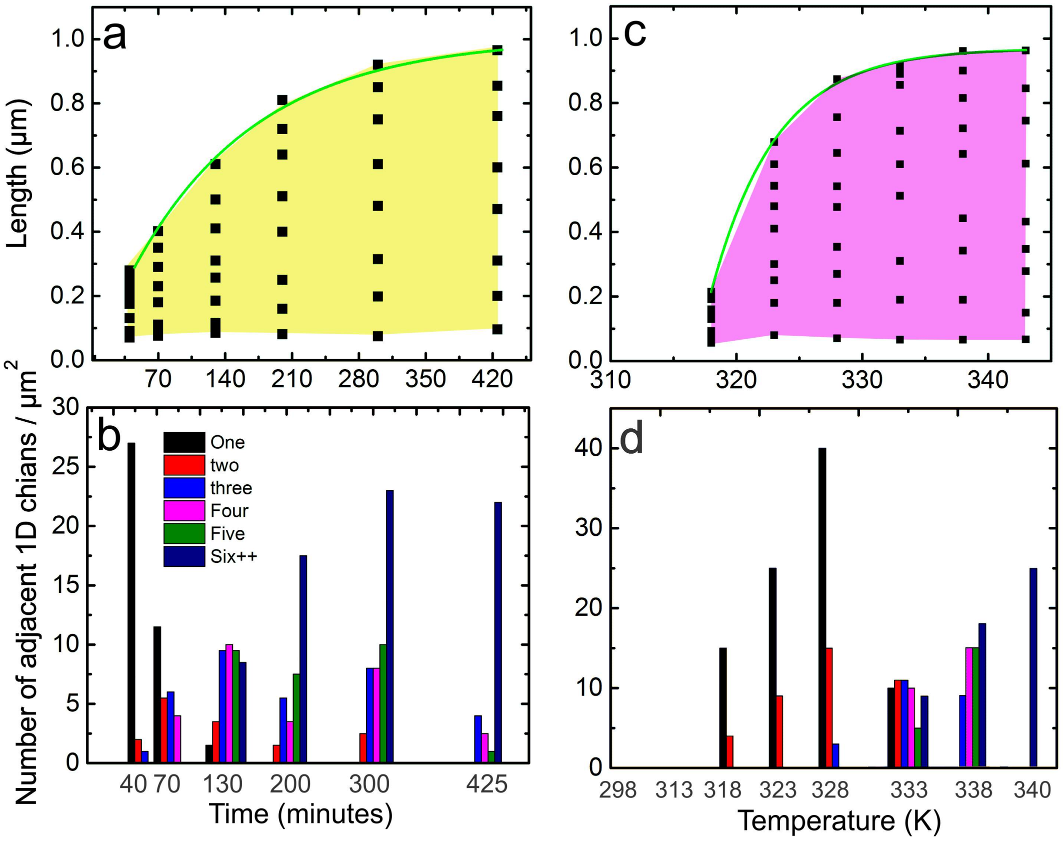

Figure 9: The statistics of the length of 1D *cis* chains (a) and 1D *trans* chains (c) for the photo– and thermal–induced switching in PABA adlayers, respectively. The statistics of the number of 1D *cis* chains (b) and 1D *trans* chains (d) for the photo– and thermal–induced switching in PABA adlayers, respectively. Black bar ("one") depicts the number of 1D chains with no neighbors.

For the photo–induces switching, except for the short illumination time, 1D chains have large number of neighbors unlike in PyABA. This suggests that the cooperativity is comparable along both molecular lattices and the nature of photo–induced switching is two dimensional (2D). However, we see that for the thermal-induced switching, at low annealing temperatures, high abundance of isolated 1D *trans* chains. That is, the thermal-induced

switching has directionality in the switching particularly at low annealing temperatures. This is also evident in the strong anisotropy in the interaction energy along different lattices of the *cis* adlayer. Green solid curves represent the exponential fit to the maximum length of the chains. Unlike in the case of PyABA, the increase in chain length is gradual for the entire range of time and temperature. This is attributed due to the comparable cooperativity along both molecular lattices and the two dimensional (2D) nature of switching. That is, domains of *cis*/*trans* is continuously formed rather than through formation of 1D chains. This also suggests that the kinetics of the switching is comparable for short and long illumination time (or for low and high annealing temperatures).

S10: The interaction energy profile for two dimers along A and B lattice directions for *trans* and *cis* PABA.

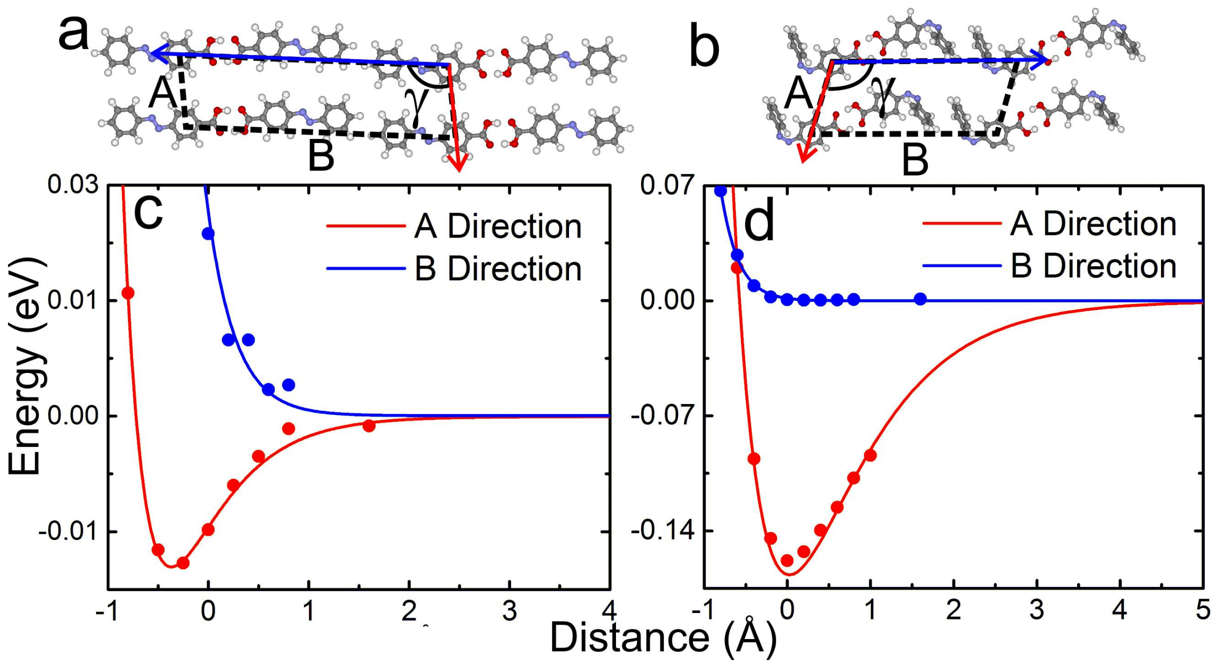

Figure 10: DFT optimized geometry of the smallest unit cell for *trans* (a) and *cis* (b) adlayers of PABA on bilayer graphite. **A**, **B** and γ are the unit lattice parameters. The interaction energy profile of adjacent dimers along the **A** (red dots) and **B** (blue dots) directions in *trans* (c) and *cis* (d) adlayers of PABA. The interaction energy is calculated without bilayer graphite. The full line curves depict the corresponding Morse potential. Zero distance marks the optimized distances of **A** and **B** on bilayer graphite.

S11: Photo(UV)-induced solution state *trans* to *cis* isomerization of PyABA and PABA in solution

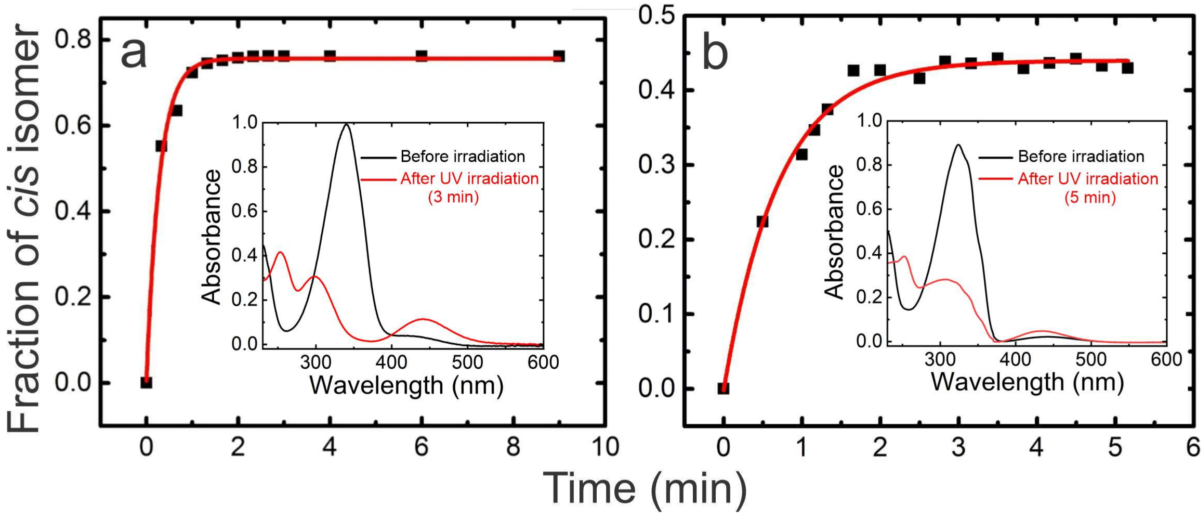

Figure 11: Photo(UV)-induced solution state *trans* to *cis* isomerization of PyABA (a) and PABA (b) in solution. The fraction of the *cis* isomer in methanolic solution of PyABA and PABA is calculated using time dependent UV-Vis spectroscopy. The peak at ≈ 441 nm (PyABA) and ≈ 435 nm (PABA) are used for the calculation of population of *cis*.

To prepare an adlayer of the *cis* isomer, solutions of PyABA and PABA were exposed to UV radiation (365 nm LED with an output power of approximately 190 mW) for 10 minutes. The proportion of *cis* isomers in the UV-irradiated solutions was determined through time-dependent photo-induced solution-state dynamics. Following UV illumination, ≈ 77% of PyABA and ≈ 44% of PABA were converted to their *cis* isomers.

S12: AFM topography of *trans* adlayer of PyABA on HOPG surface

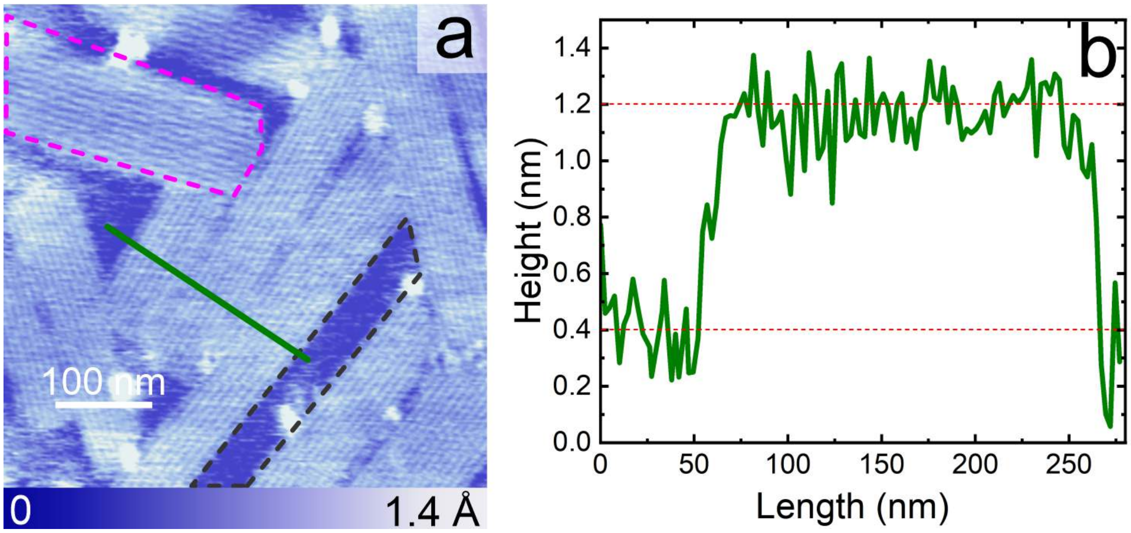

Figure 12: (a) AFM topography of adlayer of *trans* PyABA on HOPG with sub-monolayer coverage. Magenta dashed line marks a molecular island. Pristine graphite region appears as dark and is depicted by black dashed line. (b) The height profile taken along the indicated green line. The average apparent height of adlayer of *trans* isomers is ≈ 0.8 nm and is consistent to a monolayer.
